# The Effect of Real-Time Video-Based Engagement and Feedback during Pedaling on Cadence Control and Exercise Motivation: A Proof-of-Concept Study

**DOI:** 10.3390/bioengineering8070095

**Published:** 2021-07-05

**Authors:** Mukesh Soni, Tissa Wijeratne, David C. Ackland

**Affiliations:** 1 Department of Biomedical Engineering, The University of Melbourne, Melbourne, VIC 3010, Australia; mukesh.soni@unimelb.edu.au; 2 Department of Medicine and Neurology, AIMSS, Melbourne Medical School, University of Melbourne and Western Health, Sunshine Hospital, St Albans, Melbourne, VIC 3021, Australia; tissa.wijeratne@wh.org.au

**Keywords:** video feedback, exercise physiology, cycling, exercise performance, rehabilitation

## Abstract

The use of video and music as an intrinsic, dissociative attentional stimulus during exercise is thought to distract from the physical discomfort of exercise, and contribute to improved exercise adherence; however, the effects of video-based feedback and engagement during pedaling on exercise performance and motivation are poorly understood. The aims of the present study were twofold. Firstly, to develop a novel video-based engagement regime for pedaling that links pedaling cadence with the play rate of a video, and secondly, to employ an instrumented pedaling device to assess the influence of the video engagement paradigm on cadence performance and exercise motivation. Eighteen healthy subjects participated in 15-min-duration pedaling sessions while targeting a specific low cadence (60 rotations per minute) and high cadence (100 rotations per minute), including pedaling with the provision of (i) target pedaling cadence information only, (ii) visual feedback on cadence control, including pedaling duration, pedaling cadence, and cadence deviation from target, and (iii) real-time engagement, which involved pedaling at the target speed to maintain the playback rate of a pre-recorded video. Cadence deviation from the target was evaluated, and self-reported exercise motivation examined with a post-exercise survey. Pedaling-cadence deviations significantly reduced with cadence feedback at both low and high cadence (*p* < 0.05). Participants reported enjoying feedback and video-based engagement during pedaling, with 83% of participants feeling that engagement motivated them to perform pedaling-based exercise. In conclusion, real-time cadence control feedback and video-based engagement during pedaling for healthy individuals may improve performance in targeted pedaling tasks. Through dissociation from the physical cues associated with exercise and fatigue, feedback and engagement may ultimately increase enjoyment and exercise compliance and adherence of pedaling-based exercise. The findings may be useful in prescription and maintenance of targeted pedaling exercises for stroke rehabilitation and exercise therapy.

## 1. Introduction

Pedaling is an activity used in lower-limb rehabilitation and training to improve aerobic capacity and cardiopulmonary function. It provides similar periodic muscle activation patterns to those during walking [1,2,3], and since pedaling can be performed on a stationary exercise bicycle, represents a safe alternative to walking in subjects with postural instability or fall risk [1,4,5,6,7]. As a rehabilitation exercise, pedaling has also been shown to improve gait balance and motor performance in patients immediately following a stroke event [8,9]; however, the repetitive nature of pedaling on a stationary exercise bicycle is associated with poor motivation, compliance and lack of perceived self-efficacy, which are key barriers in the use of pedaling as tool in exercise therapy [10]. This is especially problematic in cases of stroke, where regular, targeted exercise therapy is integral to rehabilitation.

Objective, real-time measurement and display of exercise performance during cycling, including cadence, cadence variability, power, distance and duration of the exercise, has been shown to increase exercise performance [4,5,8,9,11,12,13,14,15,16,17]. In a study of healthy adults, visual feedback of the work done by each leg during pedaling led to higher pedaling velocities and increased gait symmetry [2]. Furthermore, in a study of stroke patients, visual feedback of electromyography (EMG), cycling cadence and torque data resulted in improvement in neuromuscular control compared to those without the feedback [5]. The use of video and music as a dissociative attentional stimulus during exercise is also thought to lower perceived exertion, distract from the physical discomfort of exercise, and contribute to improved exercise adherence [18,19,20,21,22,23]; however, the influence that dissociative stimuli such as performance feedback has on cadence control and exercise motivation during pedaling remains poorly understood.

Viewing videos during pedaling tasks has been shown to contribute to higher peak pedaling speeds for a given blood lactate level compared to pedaling without video interaction due, in part, to an altered perception of effort [24]. In a study of pedaling in women, it was shown that video and music improved the speed and distance pedalled by women without aerobic fitness training [25]. The ability of video-engagement to improve cadence performance during targeted pedaling tasks, as well as enjoyment and motivation, has received little attention in the literature to date, and may play a role in improving exercise performance and compliance in rehabilitation or exercise therapy settings. The aims of the present study were twofold. Firstly, to develop a novel video-based engagement regime for pedaling that links pedaling cadence with the play rate of a video in order to encourage exercise motivation and improve pedaling performance, and secondly, to employ an instrumented pedaling device to assess the influence of the novel video engagement paradigm as well as cadence performance feedback on real-time pedaling performance, and exercise motivation. By linking video playback with cadence performance during targeted pedaling, we hypothesize that this will improve exercise performance compared to pedaling without feedback. We hypothesize that video-based engagement and cadence control feedback during pedaling will provide a form of dissociation or distraction from bodily cues associated with fatigue and increase exercise enjoyment and motivation.

## 2. Materials and Methods

### 2.1. Subject Recruitment

Eighteen healthy individuals were recruited for testing (mean age: 27.7 years, range: 25–41 years; mean weight: 61.6 kg, range: 40–86 kg; mean height: 165.3 cm, range: 151–182 cm, 8 men and 10 women) from the University of Melbourne. The participants had no history of lower limb pain, dysfunction or previous lower-limb surgery. All were non-smokers and had varying level of physical activity in their lives (no activity: 2 participants, low/occasion: 5 participants, moderate: 9 participants, regular/daily: 2 participants). This subject number was chosen as a sample of convenience, based on a previous pedaling study of similar sample size that detected significant differences in SpO2 with exercise (Onder et al., 2013). Ethical approval was obtained from the Western Health Ethics Advisory Group (HREC/16/WH/31, HREC 2015.308), and written, informed consent was obtained.

### 2.2. Testing Apparatus

Each subject was seated at a custom-designed pedal apparatus that provided real-time cadence performance feedback and video engagement during pedaling. The pedaling apparatus comprised a seat, pedals, a cadence data recorder, and a computer with a display positioned at head-level. The seat and pedal crank position were adjusted to each subject’s lower limb lengths for optimal knee flexion and comfort. A magnet and hall-effect sensor positioned on the crank was used to measure pedaling cadence and cadence using an open-source microcontroller board (Mega2560 from Arduino, Italy), and the output validated using an external pulse generator circuit. Pedaling cadence feedback data were provided by transmitting the following data in real-time to the PC using a custom exercise monitoring program (Visual Studio 2017, Redmond, WA, USA): cycle count, pedaling cadence, duration, target cadence, and deviation from target cadence. The software program also facilitated real-time video engagement by displaying a video of the participant’s choice from a collection offered by the researchers (Appendix A). This required the participant to maintain a normal video playback rate by pedaling at the target cadence. The video playback rate was synchronised to the target pedaling cadence (intensity), and any increase or decrease from the target cadence by more than 5 rotations per minute (RPM) caused the frame rate to increase and decrease, respectively. Our pilot data showed that a smaller target range (<5 RPM) produced a task that was too challenging to achieve, while a greater target range negatively affected capacity to detect significant differences in the test groups.

### 2.3. Testing Protocol

All testing occurred at a standardised time in the afternoon (between 2 pm and 4 pm), and participants were requested not to consume any substances that may affect their performance in the 24 h prior, including alcohol or caffeine. Participants were given approximately one minute to familiarise themselves with the apparatus before testing. A series of three randomly allocated 15-min testing sessions of pedaling were then undertaken during which the subject was asked to maintain their pedaling at a target low cadence (60 RPM). Three testing conditions were established to evaluate the influence of real-time feedback of cadence and video-based engagement on cadence performance, physiology and motivation during pedaling, specifically, (i) base-line pedaling control case in which pedaling cadence feedback data were provided only in the first 20 s of each trial to allow participants to ‘establish’ their cadence, (ii) real-time visual feedback of cadence performance, including pedaling duration, pedaling cadence, and cadence deviation from target, and (iii) real-time engagement, which involved pedaling to control the playback speed of an audio-visual video (film) synchronised to the target cadence. The entire pedaling task, including the three random allocated testing conditions, was then repeated at a target high cadence (100 RPM). For all tests, the pedaling resistance was set at a moderate level, approximately half of the maximum. This was defined by a pedal load of 8.3N, producing a torque of 0.872 Nm, which was just sufficient to overcome static resistance and begin to move the crank, which was of length 10.5 cm. Rest periods of 30 s were provided every 5 min.

Pedaling cadence was recorded throughout each trial, as well as the mean-cadence deviation from target, defined by the difference between the target cadence and the mean pedaling cadence of the session. Absolute cadence deviation was evaluated and defined as the absolute difference between individual cycle cadence and target cadence. Following testing, each participant was asked to complete a survey questionnaire (File S1, Supplementary Materials) that evaluated (i) perceived differences between baseline pedaling and pedaling with feedback, (ii) overall experience with feedback, (iii) preferences in using feedback for future exercise therapy, (iv) influence of feedback on exercise motivation. The survey was completed twice for the two test conditions, once where feedback referred to real-time visual feedback of cadence data, and a second time where feedback represented real-time engagement via video playback.

### 2.4. Data Analysis

A series of two-way repeated-values analysis of variance (ANOVA) were used to assess the effect of intervention type (baseline pedaling, pedaling with feedback, pedaling with engagement) on cadence parameters while pedaling at slow and fast cadences. The cadence control dependent variables were mean-cadence deviation, coefficient of variation (CV) in mean-cadence, absolute cadence deviation and CV absolute cadence deviation. Analysis of variance was also used to assess interactions between the independent variables. Levene’s test for homogeneity of variances was performed, and a test for data normality was undertaken. Post hoc tests were undertaken using paired *t*-tests and Games–Howell tests for groups with unequal variances. CV and standard deviation were computed and used as a measure of the dispersion of data. Level of significance was defined at *p* ≤ 0.05. To demonstrate the influence of the feedback and engagement interventions during pedaling, a comparison between intervention group and baseline pedaling was drawn and effect size was computed for each variable (Appendix B).

## 3. Results

### 3.1. Cadence Control

Pedaling cadence significantly affected the absolute-cadence deviation CV (*p* < 0.001), mean-pedaling-cadence deviation (*p* = 0.032) and CV (*p* < 0.001), while intervention type had a significant influence on mean-pedaling-cadence CV (*p* = 0.005), absolute pedaling-cadence deviation, and CV (*p* < 0.001) (Table 1). There was no significant interaction between intervention type and pedaling cadence during any of the trials (*p* > 0.05). Reduction in the absolute pedaling-cadence deviation and its CV with cadence control feedback or video engagement intervention relative to baseline was dependent on pedaling cadence. For example, cadence control feedback significantly reduced the absolute pedaling-cadence deviation relative to baseline during low-cadence pedaling (mean difference: −3.0 RPM, [−4.1, −1.9 RPM], *p* = 0.028) (Table 2). A significant reduction in absolute pedaling-cadence deviation was also observed during high-cadence pedaling with cadence feedback relative to baseline (mean difference: −3.1 RPM, [−4.0, −2.2 RPM], *p* = 0.002). Similarly, a significant reduction in absolute-cadence deviation was observed with video engagement relative to baseline, during low-cadence (mean difference: −3.4 RPM, [−4.4, −2.4 RPM], *p* = 0.010) as well as during high-cadence pedaling (mean difference: −2.19 RPM, [−3.5, −0.8 RPM], *p* = 0.007). One asterisk indicates *p* ≤ 0.05, two asterisks indicate *p* ≤ 0.01, while three asterisks indicate *p* ≤ 0.001.

**Figure 1:**
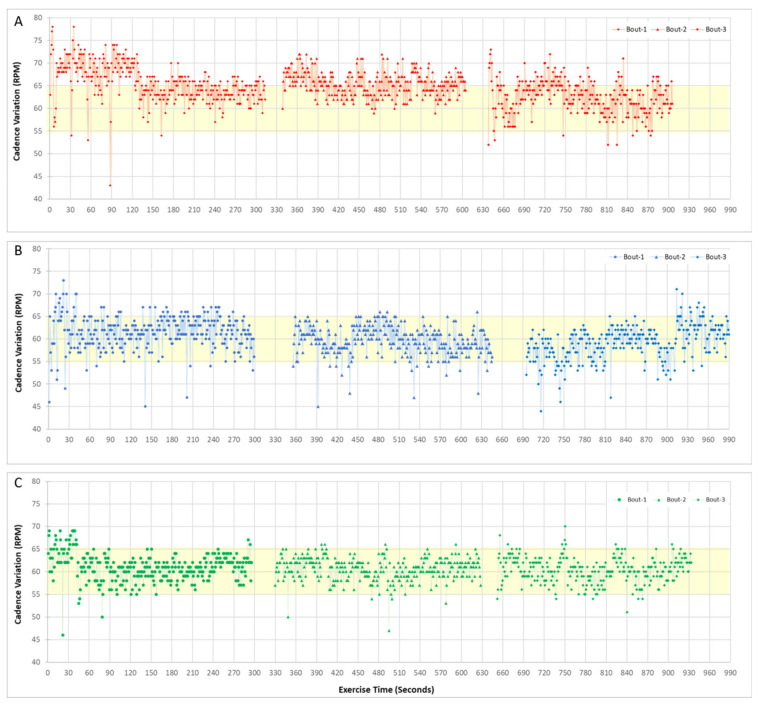
Representative time series data of pedaling cadence from one subject for baseline pedaling using a low-cadence target (60 RPM) with no feedback or engagement (**A**), real-time feedback of cadence performance (**B**) and video-based engagement (**C**). Shown are three 5-min bouts of the 15-min pedaling sessions, which included a 30 s rest break between each bout. The light-yellow shading indicates the target speed range within which the participants were asked to maintain their cadence.

**Figure 2:**
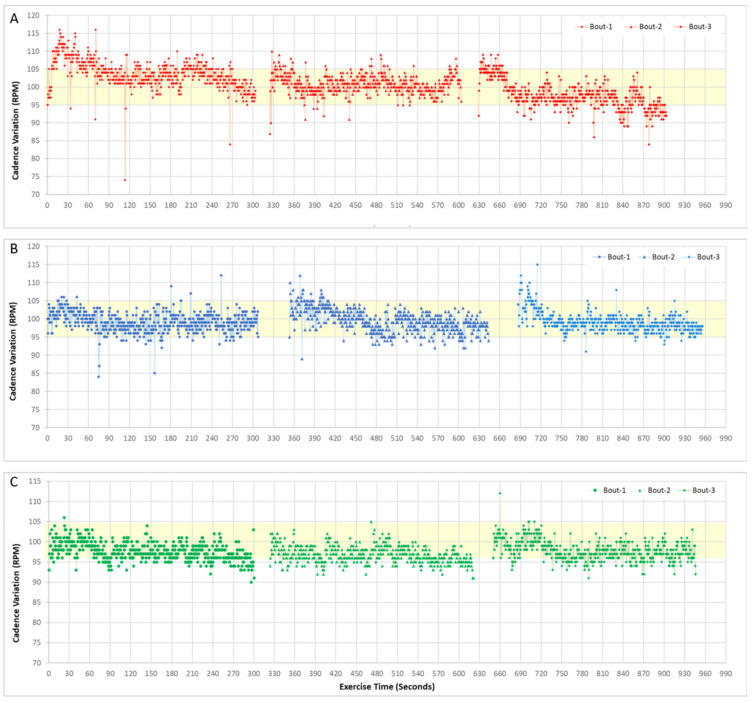
Representative time series data of pedaling cadence from one subject for baseline pedaling using a high-cadence target (100 RPM) with no feedback or engagement (**A**), real-time feedback of cadence performance (**B**) and video-based engagement (**C**). Shown are three 5-min bouts of the 15-min pedaling sessions, which included a 30 s rest break between each bout. The light-yellow shading indicates the target speed range within which the participants were asked to maintain their cadence.

The results showed that feedback and video-engagement helped the subject maintain the target 60 RPM cadence (Figure 1) (Appendix C) with video engagement resulting in an overall smoother cadence over the test duration, particularly during the second 5-min block. Similarly, during high-cadence pedaling (100 RPM), feedback and video-engagement improved pedaling control, resulting in fewer cadence variations outside of the prescribed range (Figure 2). The time series data for cadence showed that without feedback or engagement, participants were less likely to pedal consistently within the target cadence range.

The CV of the absolute-deviation in pedaling cadence during low-cadence pedaling was significantly smaller with the provision of cadence control feedback (mean difference: −0.6%, [−0.9, −0.3%], *p* = 0.029) and video engagement (mean difference: −0.8%, [−1.1, −0.5%], *p* = 0.001) relative to baseline pedaling. A similar trend was also observed during high-cadence pedaling. The CV of the absolute-deviation in pedaling cadence during low-cadence pedaling was significantly smaller with cadence control feedback (mean difference: −0.6%, [−0.9, −0.3%], *p* = 0.033) and video engagement (mean difference: −0.7%, [−0.9, −0.4%], *p* = 0.004) relative to that during baseline pedaling. Significant improvements in mean-cadence CV relative to baseline were observed with feedback (mean difference: −1.0%, [−1.5, −0.5%], *p* = 0.037) and engagement (mean difference: −0.9%, [−1.4, −0.4%], *p* = 0.034), but only during high-cadence pedaling.

### 3.2. Self-Reported Outcome Measures

**Figure 3:**
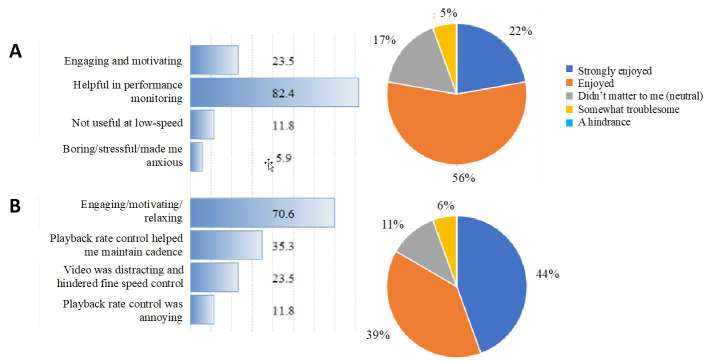
Categorization of post-exercise survey data into participant’s experience of pedaling with (**A**), feedback only compared to baseline pedaling (**B**) and video-based engagement compared to baseline pedaling.

The survey data demonstrated that 100% of participants found a difference in their exercise experience with cadence feedback and video-based engagement compared to baseline pedaling (Table S1, Supplementary Materials). 82.4% of participants found that feedback was helpful in their performance tracking compared to baseline pedaling, while video-based engagement was found to be motivating, relaxing and engaging for 70.6% of participants compared to baseline pedaling (Figure 3). Feedback during pedaling was described as engaging and motivating by 23.5% of the cohort, while 35.3% of participants found that video-based engagement helped maintain cadence control. The survey data indicated that 11.8% of participants found feedback to not be useful during low-speed pedaling, and video-based engagement to be annoying; 22% of participants strongly enjoyed the use of feedback during pedaling, while 56% of participants enjoyed the feedback. Video-based engagement was strongly enjoyed by 44% of participants and enjoyed by 39% of the cohort; 89% of participants felt that they would perform pedaling as an exercise with feedback and engagement, while 89% and 94% of participants indicated they would be motivated to perform more pedaling-based exercise in the future with the aid of feedback and video-based engagement, respectively.

## 4. Discussion

Pedaling using a stationary exercise bicycle is a popular recreational activity, and has been used in rehabilitation therapy for individuals affected by neuromusculoskeletal impairment [1,4]; however, poor motivation and compliance can have the potential to impact its effectiveness as an exercise therapy. While real-time feedback on pedaling performance has been shown to improve self-efficacy and exercise compliance [8,9], the effect of visual feedback and video engagement on cadence control and cardiovascular output during pedaling is poorly understood. The aims of the study were, firstly, to develop a novel video-based engagement regime for pedaling that links pedaling cadence with the play rate of a video in order to encourage exercise motivation and improve pedaling performance, and secondly, to assess the influence of the novel video engagement paradigm, as well as cadence performance feedback, on pedaling performance and exercise motivation. As studies have shown the dissociative influence of external audio-visual stimuli in reducing exercise-induced physiological change and improving exercise performance [26,27,28], we hypothesized that video-engagement and cadence control feedback during pedaling would yield improved pedaling performance, specifically, a smaller deviation in pedaling cadence from the target. We found that pedaling performance, including absolute deviation in pedaling cadence from a specified target cadence, as well as cadence variability, improved significantly during pedaling with cadence control feedback and video engagement when compared to baseline pedaling without feedback. This finding has implications for the use of pedaling with feedback and engagement as a therapy where targeted workloads and cadence are required to be maintained.

The post-exercise survey revealed that over two-thirds of participants found that the novel video-based engagement regime was motivating, relaxing and engaging, with 94% of participants indicating that it would motivate them to perform pedaling-based exercise therapy in the future. Pedaling on a conventional stationary exercise bicycle can be associated with low motivation and compliance [10], which may ultimately impact the effectiveness of exercise-and physical-therapy. The present study, which combined video-engagement as well as real-time visual feedback of cadence performance data, was associated with high levels of enjoyment and improved exercise motivation relative to baseline pedaling without feedback or engagement. The findings suggest that augmenting conventional pedaling therapy with performance feedback and video engagement may improve compliance and therefore the effectiveness of pedaling in applications such as rehabilitation, tele-medicine and home fitness.

In addition to improved exercise performance during pedaling, both cadence control feedback and video engagement reduced overall cadence variability (CV), irrespective of the target cadence. Similar findings have been documented in virtual reality studies, with immersive artificial environments facilitating more constant pedaling cadence, as well as increased work output and time trial performance [23,29,30]. Visual feedback during pedaling in stroke patients has also been shown to increase pedaling cadence [5]. Other studies integrating visual feedback and audio-visual stimuli to increase engagement during pedaling have documented improvements in pedaling performance [25,31]. These investigations suggest that music and video create a dissociation from the physical discomfort and fatigue associated with pedaling, resulting in the ability to produce higher intensity pedaling performance, or improvement in cardiovascular performance relative to pedaling without the stimulus.

There are limitations of this study that ought to be considered. First, a limited set of videos were provided to participants during video feedback trials, and personal preferences as well as level of stimulation may have been a confounding factor in the engagement level. A specific selection of video clips were provided to assist in standardizing the testing protocol. Second, this study assessed within-subject differences due to performance feedback and video-based engagement during pedaling, with the reference (control) state defined by pedaling without feedback or engagement. Due to the nature of the experimental design, a cohort of control subjects was not required. Future studies ought to investigate the influence of video-engagement on rehabilitation in the clinical setting, including the response of patients with varying levels of cognitive function, and whether the findings observed translate to clinically meaningful outcomes. Finally, some of the mean differences in cadence between test conditions observed in this study were small, and their clinical relevance should be interpreted with caution.

## 5. Conclusions

This study demonstrates that real-time visual feedback of cadence control as well as a novel video engagement protocol helps to improve targeted cadence pedaling performance by reducing pedaling cadence variability, which in the clinical setting, may ultimately help patients achieve targeted rehabilitation goals. These strategies may improve pedaling exercise effectiveness over a shorter time-period, though further research is warranted to quantify functional benefits, particularly over extended durations. Feedback and video-based engagement was also shown to increase exercise enjoyment and motivation. These findings suggest that feedback and video-based engagement may be useful in prescription and maintenance of targeted pedaling exercises for rehabilitation such as stroke therapy, and may ultimately help to improve exercise motivation and compliance.

## Figures and Tables

**Table 1:** Results of two-way analysis of variance (ANOVA) for cadence control parameters during pedaling at low and high cadence. The dependent variables were mean-cadence and absolute deviation from target cadence (in cycles per minute), and their coefficients of variation (%). The independent variables were intervention type (baseline pedaling, pedaling with real-time feedback of cadence performance, and pedaling with video-based engagement). Mean and standard deviation are given, as well as significant differences in intervention type (Int), pedaling cadence (Sp) and their interactions (Int*Sp) are given. One asterisk indicates *p* ≤ 0.05, two asterisks indicate *p* ≤ 0.01, while three asterisks indicate *p* ≤ 0.001.

	Low Cadence						High Cadence								
	Baseline		Feedback		Engagement		Baseline		Feedback		Engagement		*p*-Values		
	Mean	SD	Mean	SD	Mean	SD	Mean	SD	Mean	SD	Mean	SD	Int	Sp	Int * Sp
Mean cadence deviation	3.4	6.1	1.1	2.5	0.3	1.4	−0.9	6.4	−0.2	3.2	−1.0	5.8	0.187	0.032 *	0.149
CV (Mean cadence)	6.0	1.4	5.6	1.1	5.5	1.3	4.8	1.8	3.8	1.0	4.0	1.1	0.005 **	<0.001 ***	0.502
Absolute deviation	6.1	4.1	3.1	2.0	2.7	0.9	6.8	3.3	3.7	2.0	4.6	4.6	<0.001 ***	0.230	0.539
CV (Absolute deviation)	4.5	0.9	3.9	1.0	3.7	0.9	3.4	0.9	2.8	0.8	2.8	0.6	<0.001 ***	<0.001 ***	0.845

**Table 2:** Comparison of cadence parameters during pedaling at low and high cadence. The means are compared between intervention types (baseline pedaling, pedaling with real-time feedback of cadence performance, and pedaling with video-based engagement) in pairs at both the pedaling cadences. Mean difference and their significance (p value) are given, as well as 95% confidence intervals and their lower limit (LL) and upper limit (UL). One asterisk indicates *p* ≤ 0.05, two asterisks indicate *p* ≤ 0.01.

	Low Cadence												High Cadence											
	Feedback vs. Baseline				Engagement vs. Baseline				Engagement vs. Feedback				Feedback vs. Baseline				Engagement vs. Baseline				Engagement vs. Feedback			
	Mean Diff.	*p* Value	95% CI		Mean Diff.	*p* Value	95% CI		Mean Diff.	*p* Value	95% CI		Mean Diff.	*p* Value	95% CI		Mean Diff.	*p* Value	95% CI		Mean Diff.	*p* Value	95% CI	
			LL	UL			LL	UL			LL	UL			LL	UL			LL	UL			LL	UL
Mean cadence	−2.3	0.338	−3.8	−0.7	−3.0	0.130	−4.5	−1.6	−0.8	0.500	−1.5	−0.1	0.7	0.607	−1.0	2.4	−0.1	0.939	−2.1	1.9	−0.8	0.471	−2.4	0.7
CV (mean cadence)	−0.4	0.166	−0.9	−0.0	−0.6	0.083	−1.0	−0.1	−0.1	0.687	−0.5	0.3	−1.0	0.037 *	−1.5	−0.5	−0.9	0.034 *	−1.4	−0.4	0.1	0.582	−0.2	0.5
Absolute cadence deviation	−3.0	0.028 *	−4.1	−1.9	−3.4	0.010 **	−4.4	−2.4	−0.4	0.723	−0.9	0.1	−3.1	0.002 **	−4.0	−2.2	−2.2	0.007 **	−3.5	−0.8	0.9	0.383	−0.3	2.1
CV (absolute cadence deviation)	−0.6	0.029 *	−0.9	−0.3	−0.8	0.001 **	−1.1	−0.5	−0.2	0.346	−0.5	0.1	−0.6	0.033 *	−0.9	−0.3	−0.7	0.004 **	−0.9	−0.4	−0.1	0.644	−0.3	0.2

**Figure A1:**
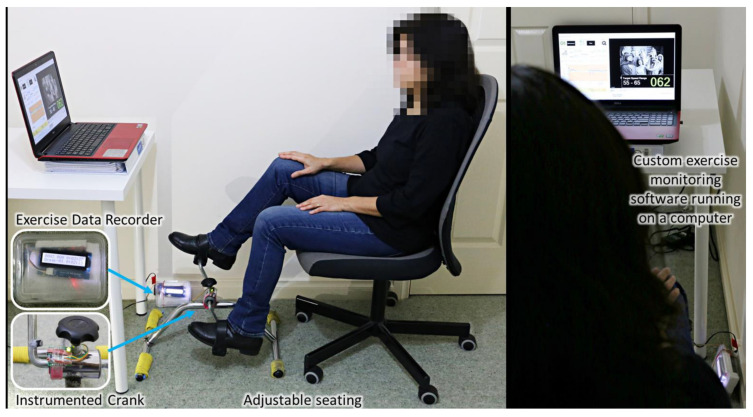
Experimental setup used for pedaling trials. A custom-designed pedaling apparatus was developed comprising an instrumented crank, pedals, an electronic exercise data recorder to measure pedaling cadence, custom software running on a computer, and a seat with adjustable height.

**Figure A2:**
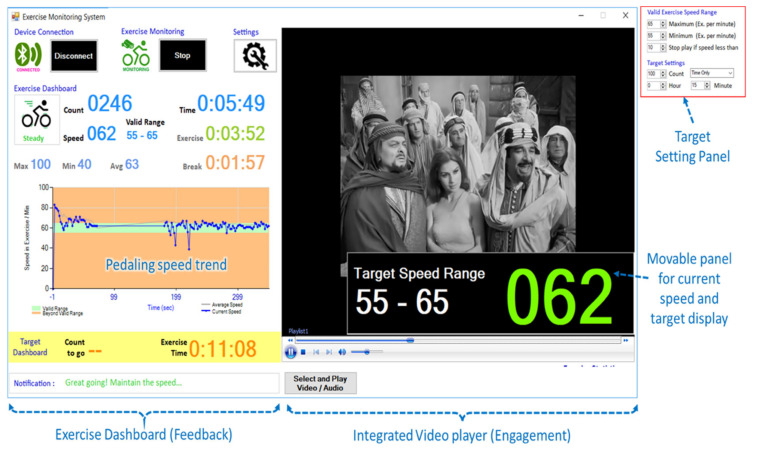
Graphic user interface (GUI) of software employed during pedaling trials. The GUI provided feedback on exercise performance using a smart ‘dashboard’ on the left half of the screen. The right half of the screen contained an integrated video/music player to provide engagement during exercise. An optional floating window was used to configure pedaling cadence settings.

## Data Availability

Not applicable.

## Supplementary Materials

The following are available online at https://www.mdpi.com/article/10.3390/bioengineering8070095/s1, Table S1: Post-exercise feedback survey results for questions 1, 3 and 4, File S1: survey questionnaire.

## Appendix A

### Custom Cycle Ergometer

A commercially available stationary pedaling crank (Standard Pedal Exerciser, Chattanooga Group, Austin, TX, USA) was modified with sensor electronics to record pedaling cadence during test sessions (Figure A1). A magnet attached to the pedal crank triggered a hall-effect sensor (DRV5013ADQLPGMQ1, Texas Instruments) to generate one pulse every complete cycle revolution. These pulses were captured, and the timing information processed using microcontroller-based electronic hardware to compute cadence (Mega2560, Arduino, Somerville, MA, USA). The instantaneous cadence, cycle count and other information related to exercise timing were captured and transmitted to a desktop PC-based hardware (Windows 7+, Intel core i7, 8GB RAM) over Bluetooth (HC-06 module).

The graphic user interphase (GUI) used during testing included controls for connecting with the electronic hardware via USB (wired) or Bluetooth (wireless) and receiving the exercise data transmitted from the hardware (Figure A2). An exercise dashboard on the software displayed the exercise information along with a graph of instantaneous cadence with respect to time. Additionally, a video player was displayed which allowed participants to watch a video from the computer during the exercise. The video playback was linked with pedaling cadence using a configuration panel. The video playback rate could be synchronised to the target pedaling cadence, and for any increase or decrease from the target cadence by a specific amount, the frame rate proportionally increased and decreased, respectively.

## Appendix B

### Appendix B.1. Effect Size

Effect Size is a direct indicator of overlap between the two sample groups in terms of a comparison of percentiles (Table A1) and is computed as the ratio of the mean difference between baseline and intervention group to the standard deviation.

$$Effect\;Size=\frac{Abs\left(Mean_{Intervention\;Group}-Mean_{Baseline}\right)}{Standard\;Deviation_{Baseline}}$$

### Appendix B.2. Interpretation of the Effect Size

We used effect size to quantify the size of the difference between intervention groups (visual feedback and video engagement), relative to baseline pedaling (without feedback or engagement). For example, an effect size of 0.9 during high-cadence pedaling indicated that the absolute deviation with visual feedback was smaller than 82% of participants during baseline pedaling, on average (Table A2).

**Table A1:** Interpretation of study effect size. The magnitude of the comparison quantity between the control and experimental groups is given as the effective size. Conversions of effect size (column 1) to percentile (column 2), probability of correctly identifying a person’s group from score (column 3) and binomial effect size display (BSED on column 4) are shown.

Effect Size	Percentage of Control Group below the Average Individual in the Test Group	Probability That One Could Guess Which Group a Person Was in from Knowledge of Their ‘Score’	Probability That an Individual from the Test Group Is Higher Than an Individual in the Control Group, If Both Chosen at Random
0.0	50.0%	0.50	0.50
0.1	54.0%	0.52	0.53
0.2	58.0%	0.54	0.56
0.3	62.0%	0.56	0.58
0.4	66.0%	0.58	0.61
0.5	69.0%	0.60	0.64
0.6	73.0%	0.62	0.66
0.7	76.0%	0.64	0.69
0.8	79.0%	0.66	0.71
0.9	82.0%	0.67	0.74
1.0	84.0%	0.69	0.76
1.2	88.0%	0.73	0.80
1.4	92.0%	0.76	0.84
1.6	95.0%	0.79	0.87
1.8	96.0%	0.82	0.90
2.0	98.0%	0.84	0.92
2.5	99.0%	0.89	0.96
3.0	99.9%	0.93	0.98

**Table A2:** Effect size of the pedaling-based interventions on cadence parameters at low and high cadence, relative to baseline. The dependent variables were mean-cadence (RPM), absolute deviation of cadence from target cadence (RPM), and their coefficients of variation (%). The independent variables were intervention type (baseline pedaling, pedaling with feedback, and pedaling with engagement). Mean and standard deviation are given, and significant differences in intervention type (Int), pedaling cadence (Sp) and their interactions (Int*Sp) are shown. One asterisk indicates *p* ≤ 0.05, two asterisks indicate *p* ≤ 0.01, while three asterisks indicate *p* ≤ 0.001.

	Low Cadence						High Cadence								
	Baseline		Feedback		Engagement		Baseline		Feedback		Engagement				
	Mean	SD	Mean	SD	Mean	SD	Mean	SD	Mean	SD	Mean	SD	Int	Sp	Int * Spa
Mean cadence deviation	3.4	6.1	1.1	2.5	0.3	1.4	−0.9	6.4	−0.2	3.2	−1.0	5.8	0.187	0.032 *	0.149
CV (Mean cadence)	6.0	1.4	5.6	1.1	5.5	1.3	4.8	1.8	3.8	1.0	4.0	1.1	0.005 **	<0.001 ***	0.502
Absolute deviation	6.1	4.1	3.1	2.0	2.7	0.9	6.8	3.3	3.7	2.0	4.6	4.6	<0.001 ***	0.230	0.539
CV (Absolute deviation)	4.5	0.9	3.9	1.0	3.7	0.9	3.4	0.9	2.8	0.8	2.8	0.6	<0.001 ***	<0.001 ***	0.845

## Appendix C

### Mean Cadence

**Table A3:** Mean cadence during low- and high-cadence pedaling for baseline pedaling, pedaling with real-time feedback of cadence performance, and pedaling with video-based engagement. Data are given and RPM, and standard deviation are also provided.

		Baseline		Feedback		Engagement	
		Mean	SD	Mean	SD	Mean	SD
Low Cadence		63.4	6.1	61.1	2.5	60.3	1.4
High Cadence		99.1	6.4	99.8	3.2	99.0	5.8
