# Supplementary material for: The Effect of Real-Time Video-Based Engagement and Feedback during Pedaling on Cadence Control and Exercise Motivation: A Proof-of-Concept Study
Source: Bioengineering (Basel). 2021 Jul 5;8(7):95. doi: 10.3390/bioengineering8070095 (PMC8301120; doi:10.3390/bioengineering8070095)
Supplement: Supplementary file 1 [file bioengineering-08-00095-s001.zip › bioengineering-1248365-supplementary.pdf]

SUPPLEMENTARY MATERIAL

Exercise Feedback Survey

1. Did you notice a difference between exercise sessions when you received feedback compared to when you didn't?
   - No
   - Yes. The difference could be described as ______________________________________
   ____
   ____

2. If you found the two sessions different, how would you describe the session in which feedback was provided?
   - Strongly enjoyed
   - Enjoyed
   - It didn't matter to me (neutral)
   - Somewhat troublesome
   - A hindrance

3. In future, would you choose to perform exercise with feedback?
   - Yes
   - No
   - Undecided

4. In future, would feedback encourage you to exercise more?
   - Yes
   - No

Table S1: Post-exercise feedback survey results for questions 1, 3 and 4.

	Feedback		Engagement	
	Yes	No	Yes	No
Did you find a difference between excerise sessions when you received feedback compared to when you didn't?	100%	0%	100%	0%
In future, would you choose to perform exercise with feedback?	89%	11%	89%	11%
Would feedback encourage you to exercise more?	89%	11%	94%	6%
